# Protective Ferments

**Published:** 1913-12

**Authors:** I. Walker Hall

**Affiliations:** Professor of Pathology, University of Bristol, and Pathologist to the Bristol Royal Infirmary.


### PROTECTIVE FERMENTS.

BY

I. Walker Hall, M.D. Vict.

*Professor of Pathology, University of Bristol, and Pathologist to the Bristol Royal Infirmary.*

---

The tissues are protected from the entry of heterologous proteins by action of the digestive apparatus. The food enters the body proper, except during gastro-intestinal catarrh or ulceration, in a form suitable for the capacities of the various types of cellular metabolism; namely, as amino-acids, or altered proteins, etc. The further building up, or breaking down, of these substances depends upon the characters of individual cells. The means employed for this purpose are strictly specific. For instance, it has just been shown that the blood serum of a dog will hydrolyse muscle protein derivatives (peptone), but not the proteins, of dog's muscle, or of cat's muscle, and *vice versa*. There are, however, group reactions, for fox serum hydrolyses dog's muscle peptone, and dog's serum hydrolyses fox muscle peptone.

When heterologous proteins or other food substances reach the tissues without being previously broken down or changed into homologous proteins, they are regarded apparently as of the nature of foreign bodies, and methods are evolved for their removal. The most reasonable way by which they can be prepared for removal is along the lines of ordinary alimentary digestion. The substances which effect this "protective" type of digestion do not occur normally in the tissue cells themselves. There is a sufficiency of substances for the cleavage of amino-acids, etc., that is to say, peptolytic ferments, but neither tryptic nor peptic enzymes. What is to be done? The tissues have to rise to the emergency, and produce the means for dealing with the foreign body in question. Some days elapse before the necessary capacity for bringing this about is attained. A similar delay occurs, it will be remembered, in connection with the appearance of such antibodies as agglutinins and lysins.

The presence of these new powers is manifested by a change in the characters of the blood serum. This change may be demonstrated in several ways. In one, purified albumins are prepared from the foreign proteins: serum containing specific ferments is then allowed to act upon the pure albumin, and the resultant products are separated by dialysis and subsequently identified. In another method, peptones derived from the heterologous protein are examined in a special form of polariscope, and the deviations read from time to time. Both methods are at present in process of refining, and much care is needed if more or less obvious fallacies are to be avoided. The technical difficulties are discussed in the references appended.

One of the practical applications of this experimental work, carried out in Berlin and Halle at the instigation of Abderhalden, is a test designed to assist in the diagnosis of pregnancy during the first months. Schmorl has shown that the placenta allows the transmission of chorionic cells, or placental albumin, into the maternal blood stream. The albumin is neither broken down into amino-acids nor re-constituted into homologous albumin, so that it circulates as a foreign albumin in the blood stream, and acts as a stimulus to the formation of "protective" ferments. These ferments are produced in due course, and may be demonstrated in the blood stream as early as at the end of the first month. It should be borne in mind, however, that these ferments are anti-placental and not anti-fœtal. The test tells, therefore, whether placenta is present or not. The results accumulated from sources all the world over, including a fair number from Bristol during 1912 and 1913, go to show that about ninety-five per cent. of pregnant and one per cent. of non-pregnant women yield serum giving a positive reaction. Carcinoma and fever seem to interfere with the sharpness of the reaction. One case is reported in which blood serum obtained from a male gave a positive reaction : whether this was a case of suspected chorio-epithelioma is not stated. The reaction has been found negative in two cases of placental polypi and retained placental tissue. The position appears to be as follows. A serum test for pregnancy is on its trial, its technique will most probably be improved as time goes on. At present it may be regarded as a useful adjuvant to clinical signs, and may afford aid also in the differentiation of pregnancy from tumours of the uterus and adnexa. It may with advantage be associated with an estimation of the urinary creatine, which shows an increase very early in pregnancy. It may help to distinguish between slight and severe cases of eclampsia, and may even afford some indication of its onset. Apart from the difficulties of the technical procedures, there are some fallacies connected with the collection of the blood. The serum should be free from hæmoglobin if the ultimate reading is to be definite. It must, therefore, be collected very gently, and not disturbed during the process of coagulation. A stay of four hours in an ice chest is preferable. 5 c.c. of blood are necessary, and he upper layers of the serum are used after repeated centrifugalisation. The test must be carried out before the blood has become old, for if any decomposition has taken place, and ammonia has been formed, the results are of little value.

Another application relates to the presence of specific "protective" ferments in the blood serum in certain diseases. For instance, Fausser states that in *dementia præcox* the patient's serum is able to break down testicular and brain tissue. In syphilis and para-syphilis a similar action upon coagulated nerve proteins is present. In thyroid lesions there is a cleavage of thyroid albumin, although this is less marked.

In thirteen cases of persistent thymus the blood serum formed peptones from albumins prepared from thymus tissue, and thus indicated the presence of status lymphaticus and lymphatism, confirmed in some of the instances by operation or autopsy (Bauer). From the control standpoint, Lampè has shown that normal serum does not alter proteins prepared from thyroid or thymus, or liver, or pancreatic, or supra-renal, or ovarian, or testicular, or muscular tissue.

If albumins are prepared from atrophic musclc fibres and serum obtained from patients suffering from muscular atrophy in cases of cornual changes, or disseminated sclerosis or of tabes, s allowed to act upon them, peptones are formed from the albumins.

In pneumonia it has been found that the blood contains ferments which act specifically upon albumins prepared from the pneumococcus. The feature is most evident at the period of crisis, and may have something to do with its onset.

A considerable amount of work is now being carried out in order to determine whether the blood serum of carcinomatous individuals possesses protective ferments against cancer cells; whether the presence of lymphadenoma may be similarly diagnosed, and so on. It is quite possible that the inhibitory effects of carcinomatous extracts upon the rate of division of paramecium may be due to substances of an allied nature.

The field for further investigation is a very large one. So long as it is left to those who are working at it, well and good. It is not yet ready for the casual labourer. The outlook is full of promise, and the next two years may witness remarkable developments. In the meantime, our standpoint must perforce be embodied in the popular catchword, "wait and see!"

### REFERENCES.

[1] E. Abderhalden, *Schutzfermente des tierischen Orgamismus*, Berlin, 1912, and numerous papers in *Ztschr. f. physiol. Chem.*, 1913.

[2] Gottschalk, *Berl. klin. Wchnschr.*, 1913, l. 1151.

[3] Lampè, *München. med. Wchnschr.*, 1913, lx. 1423.

[4] Bauer, *Wien. klin. Wchnschr.*, 1913, xxvi. 606, 1109.

[5] Frank, *Berl. klin. Wchnschr.*, 1913, l. 906.

[6] Veit, *Ztschr. f. Geburtsh.*, 1912, lxxii. 463.

[7] Fausser, *Deutsche. med. Wchnschr.*, 1912, 52.

[8] Walker Hall and J. W. Taylor, Walker Hall and Scott-Williamson, *J. Path. and Bacteriol.*, 1911, xv. 350; 1912, xvii. 121.

[9] Woodruff and Underhill, "Protozoan Protoplasm as an Indicator of Pathological Changes," *J. Biolog. Chem.*, 1913, xv. 385.

[10] Papazola, *C. R. Soc. Biol.*, 1913, Bd. 74, No. 16.

